# Hepatitis C Viral Infection Among Beta-Thalassemia Patients: A Study From the Centre for Excellence in Thalassemia and Other Blood Disorders

**DOI:** 10.7759/cureus.16207

**Published:** 2021-07-06

**Authors:** Venkataramana Kandi, Sravani Reddy Vinjamuri, Bhanu Pravallika Tanikella

**Affiliations:** 1 Clinical Microbiology, Prathima Institute of Medical Sciences, Karimnagar, IND; 2 Medicine, Prathima Institute of Medical Sciences, Karimnagar, IND

**Keywords:** beta-thalassemia, hepatitis c virus, blood transfusions, chronic hepatitis, blood disorders, infection, prevention

## Abstract

Background

Hepatitis C virus (HCV) is a single-stranded RNA virus, which is frequently transmitted through blood transfusions, contact with infected blood or blood products, and vertical transmission. Injectable drug abusers and transplant recipients are predisposed to HCV infection. It causes acute hepatitis, which may progress to chronic hepatitis, and in severe untreated cases, patients may develop cirrhosis and hepatocellular carcinoma (HCC). Since there is no vaccine available against HCV infection, prevention remains the mainstay, at least among the susceptible populations that include thalassemia patients.

Methods

A prospective case-control study was conducted at the center for excellence in thalassemia and other blood disorders attached to the Prathima Institute of Medical Sciences (PIMS), a tertiary care teaching hospital at Karimnagar, Telangana, India. Blood samples of 100 beta-thalassemia patients and age-matched non-thalassemic persons were screened for antibodies against HCV by an enzyme-linked immunosorbent assay (ELISA) based rapid immunochromatographic method, and the chemiluminescence assay using the Abbott AxSYM (Abbott Laboratories, Abbot Park, IL, USA). During the same period, the prevalence of HCV was assessed among non-thalassemic patients attending in-patient and out-patient wards of PIMS hospital.

Results

Of the 100 cases of beta-thalassemia, 28 (28%) were HCV positive. All the age-matched non-thalassemic controls were negative for HCV antibodies. Among the positives, 20 (71%) were males, and eight (29%) were females. The prevalence of HCV among non-thalassemic patients attending the hospital during the same period was found to be 0.19%.

Conclusions

HCV infection among the beta-thalassemia patients was abnormally high as compared to the others. Thalassemia patients are potentially predisposed to HCV infection and other blood-borne viral infections. Thorough screening of blood before transfusion is warranted. HCV infection may further increase the morbidity and mortality of beta-thalassemia patients and other patients with blood disorders who acquire the infection due to frequent blood transfusions.

## Introduction

Hepatitis C Virus (HCV) is an RNA virus that belongs to the genus *Hepacivirus *and family *Flaviviridae*. HCV is an enveloped virus, spherical in shape, and has a positive sense (+) RNA [1]. The prevalence of HCV is reported to be extremely high among hemodialysis patients, Human immunodeficiency virus (HIV) infected people, and others who have a history of multiple blood transfusions [2,3]. HCV infections are generally chronic, and most exposures go unnoticed. Also, the HCV disease course remains silent wherein the patients develop intermittent symptoms including mild jaundice and abnormalities in the liver enzymes. The virus remains confined to the liver cells and only enters the bloodstream intermittently. Therefore, the diagnosis of HCV infection is often missed by routine blood tests. The infection may be transmitted by several routes that include sharing needles (injectable drug users), unprotected sex, transplacental transmission from mother to child, hemodialysis, and transfusion of blood and blood products [1]. Among the different modes of transmission, repeated blood transfusions, and hemodialysis is found to predispose people to HCV infection [4]. Active HCV infection could contribute to an additional burden on the patients suffering from beta-thalassemia.

Beta thalassemia is an inheritable genetic disorder that affects the synthesis of the β-globin chain of the hemoglobin molecule. Thalassemia presents as severe anemia among the affected patients who require frequent blood transfusions [5]. Among several other complications arising from repeated blood transfusions, thalassemia patients are predisposed to bloodborne microbial infectious diseases. This may further compromise the health, well-being and increase the morbidity and mortality of thalassemia patients. Because there is no vaccine against HCV infection, prevention of transmission remains the mainstay while managing beta-thalassemia patients [6]. Also, important is to understand the adverse effects of antiviral therapy on patients. Therefore, careful screening of blood and blood products against HCV assumes increased significance. 

The present study is carried out to evaluate the prevalence of HCV infection among the beta-thalassemia patients attending the government-accredited center for excellence in thalassemia and other blood disorders attached to a tertiary care teaching hospital in North Telangana

The results of this study were previously presented at the International Science Symposium on HIV and Infectious Diseases (ISSHID 2019), Chennai, India, 12-14 October 2019, and the published abstract is available at https://bmcinfectdis.biomedcentral.com/articles/10.1186/s12879-020-05038-y#Sec226.

## Materials and methods

This prospective case-control study was done between January and June 2019 and included 100 patients diagnosed as suffering from beta-thalassemia. Age-matched non-thalassemic persons (n=100) were included as the control group. A total of 4153 non-thalassemic patients attending Prathima Institute of Medical Sciences (PIMS) during the same period were also assessed for the presence of anti-HCV antibodies. 

All the thalassemic patients recruited were belonging to the age group between five and 15 years. The thalassemia patients were treated as a part of a national program at the center for excellence in thalassemia and other blood disorders attached to PIMS, a tertiary care teaching hospital, Telangana, India. An informed and oral consent was obtained from the parents/guardians of the subjects because they belonged to pediatric age. The institutional ethics committee clearance of the PIMS (IEC/PIMS/2019-004-01092019) was obtained for the conduction of the study. 

The inclusion criteria for the cases were a confirmed diagnosis of beta-thalassemia, and the control group was non-thalassemic. All patients who had other co-infections were excluded from the study. 

Three milliliters of blood were collected from each patient, and after separating the serum, a fourth-generation HCV TRI-DOT (Diagnostic Enterprises, H.P., India) kit was used for the qualitative detection of HCV-specific antibodies and a chemiluminescence assay was performed using Abbott AxSYM (Abbott Laboratories, Abbot Park, IL, USA) instrument for quantitative evaluation of anti-HCV antibodies. The test results were interpreted as per the manufacturer’s instructions.

The data were entered into a Microsoft Excel sheet, percentages were calculated and tables were generated.

## Results

Of the 100 beta-thalassemia patients tested, 28 (28%) were found to be positive for anti-HCV antibodies. All the age-matched non-thalassemic persons were negative for anti-HCV antibodies. Among the beta-thalassemia patients positive for anti-HCV antibodies 20 (71%) were males and eight (29%) were females, as shown in Table 1.

**Table 1:** Anti-HCV antibodies among the beta-thalassemia patients HCV: Hepatitis C Virus

Gender	Total cases n (%)	Cases positive for anti-HCV antibodies n (%)
Male	57 (57%)	20 (71%)
Female	43 (43%)	8 (29%)
Total	100 (100%)	28 (28%)

Of the 4153 non-thalassemic patients attending the PIMS hospital during the study period who were tested, eight (0.19%) were positive for anti-HCV antibodies. The comparative rates of anti-HCV antibody seropositivity of the cases, controls, and hospital patients are depicted in Table 2.

**Table 2:** Comparison of anti-HCV antibody positivity among thalassemic patients, non-thalassemic controls, and hospital patients HCV: Hepatitis C Virus

Test	Beta-thalassemia patients/cases (n=100)	Age matched non-thalassemic controls (n=100)	Non-thalassemic patients attending hospital (n=4153)
Anti-HCV antibody	28 (28%)	0 (0%)	8 (0.19%)

## Discussion

HCV is one among the hepatitis viruses that also include Hepatitis A (HAV), B (HBV), D (HDV), and E (HEV) viruses among others [7]. HCV and HBV cause serum hepatitis and are transmitted by the parenteral route. HAV, and HEV cause infectious hepatitis that is transmitted to humans by the feco-oral route. HDV was found to exist along with HBV and its role in causing hepatitis is not completely understood. Both HBV and HCV are associated with acute and chronic infections, whereas HAV and HEV are responsible for self-limiting acute hepatitis. 

More than 80% of the HBV and HCV infections resolve without any specific treatment; whereas, in some people, the exposure could lead to the chronic carriage of the virus. During chronic hepatitis caused by HBV and HCV, the virus establishes itself in the hepatocytes and only frequently invades the bloodstream causing symptomatic episodes in infected people. This gradually leads to liver damage that presents as liver cirrhosis, and in some people, hepatocellular carcinoma (HCC) can develop [8]. 

It is now a fact that HBV is more infectious than HCV, wherein household contacts could potentially acquire HBV infection. In contrast, HCV infection generally occurs in people who have a history of multiple blood transfusions and does not spread by ordinary contact. Nevertheless, transmission within the family members is a possibility when sharing razors, contact with blood, and constant exposure to blood and body fluids [9].

A high prevalence of HCV was noted in thalassemia patients, hemodialysis patients, and injectable drug users, among others [10]. Given the increasing prevalence of thalassemia in India and several parts of the world, there is an urgent need to focus on the prevalence of HCV among such populations [11]. HCV-infected thalassemia patients may suffer from hastened disease progression and the antiviral therapy against HCV can cause increased morbidity and compromise the quality of life of thalassemia patients [12]. Because there is no vaccine available for the prevention of HCV infection, unlike against HBV, prevention of infection, and its spread appears to be of great significance. 

As noted by the results of the present study, the prevalence of HCV among the thalassemia group (28%) was extremely high as compared to the age-matched non-thalassemic control group (0%), and patients presenting to the hospital (0.19%) for various reasons. 

HCV co-infection among HIV-infected people (2.4%), pregnant women (4.0%), homosexual groups (6.4%), and drug addicts (82.4%) reveal high prevalence as compared to the burden among the general population [13]. A region-wise HCV prevalence among homosexual men revealed that the African (5.8%) and the South-East Asian (5.0%) groups had the highest rates. HCV among HIV seropositive homosexuals (6.22%) was considerably higher than the HIV seronegative homosexuals (1.58%) [14].

Less than 1% prevalence was noted among blood donors, pregnant women, and community studies. HCV prevalence in the high-risk groups was variable and alarmingly high among HIV patients (3.5%), hemodialysis (19.2%), injectable drug addicts (44.71%), persons who underwent multiple blood transfusions (24.0%), and people with abnormal sexual behavior (4.2%) [15].

An assessment of HCV incidences in America between 2013 and 2016 revealed a 1.7% prevalence [16]. HCV prevalence among the Egyptian population was noted to be above 2% with people with HEV co-infection and advanced liver disease showing quick progression to liver failure and HCC [17]. 

HCV genotypes prevalent among Indian drug abuse population were 3a (39%), 3b (20.7%), and 1a (26.9%) among others [18]. Prevalence in India ranges between 0.5% and 1.5% and the state of Punjab reported a high prevalence of >3.2% [19,20]. HCV prevalence among the tribal population of Eastern parts of India was high (>6%) due to a lack of education concerning the mode of transmission of infection [21]. 

The prevalence of HCV among eye donors was noted to be 0.52% as reported from Chennai, India [22]. Febrile patients attending a tertiary care hospital reported a 2.3% HCV prevalence, Gujarat, West part of India [23]. Prevalence of 1.06% in hospitalized patients from a study in West Bengal, Eastern part of India [24]. 

Prevalence of HCV among hemodialysis patients attending a tertiary care hospital in South India revealed 9% (1% in antibody testing and 8% in RNA detection) [25]. Hemodialysis patients showed an 18.8% prevalence from a study from Pune, India [26].

HCV prevalence among the thalassemia patients was found to be extremely high (59.3%) as noted by a study from Orissa, India [27]. HCV prevalence was 51.1% among thalassemia patients as reported from Surat, Gujarat, Western India [28]. The prevalence of HCC among thalassemia was found to be 1.66% and fractionally higher among transfusion nondependent HCC in thalassemia patients (1.96%) [29]. A study from Oman reported 41% HCV prevalence among thalassemia patients [30].

Limitations of the study

The present study has focussed on the prevalence of HCV among thalassemia patients, and therefore, several other clinical aspects like how the patients were managed after the diagnosis, factors responsible for the high rates of infection, and others were not addressed in this study. 

Future implications of the study

The results of this study and several others from various geographical regions have noted an extremely high prevalence of HCV infection among thalassemia patients. Further to this study, there is an urgent need to improve blood transfusion practices to avoid the transmission of bloodborne infectious diseases that include HCV, among others. Given the emerging pieces of evidence of HCV being potentially transmissible among household contacts, frequent screening of thalassemia patients, educating them and their families appear increasingly important. 

## Conclusions

HCV prevalence is remarkably higher among thalassemia patients. HCV infection among the general population and non-thalassemic patients attending hospitals was considerably low. The results of the current research study and the available literature indicate potential patient groups including those suffering from thalassemia are increasingly predisposed to HCV infections and therefore could act as reservoirs of infection. Given the absence of an approved vaccine, extensive screening, prevention, and management programs are required to control the spread of HCV infection.
